# Perceptions, awareness and influences of medical students towards plastic surgery: A systematic review

**DOI:** 10.1016/j.jpra.2024.04.003

**Published:** 2024-04-08

**Authors:** Sevasti Panagiota Glynou, Christina Anna Petmeza, Ariadni Georgiannakis, Sara Sousi, Alexander Zargaran, David Zargaran, Afshin Mosahebi

**Affiliations:** a Barts and the London School of Medicine and Dentistry, Queen Mary University of London, London, UK; b Department of Medicine, Imperial College London, London, UK; c Department of Plastic Surgery, University College London, London, UK; d Royal Free Hospital, London, UK

**Keywords:** Plastic surgery, Medical education, Perceptions

## Abstract

**Introduction:**

Plastic, reconstructive and aesthetic surgery (PRAS) is a significant yet often overlooked specialty in medical school curricula. The impact of social media and unregulated information sources can distort the perceptions of medical specialties, including PRAS, leading to a decline in student interest, inappropriate referrals and strain on healthcare services. This systematic review aimed to understand the perceptions of medical students towards PRAS, identify influencing factors and explore strategies to address these influences.

**Methods:**

The review followed the PRISMA 2020 guidelines. Four databases were searched, and the inclusion and exclusion criteria were applied. Data from 17 relevant studies were analysed in Microsoft Excel using descriptive statistics. The risk of bias was assessed using a modified Newcastle–Ottawa Scale.

**Results:**

Medical students generally held positive perceptions about PRAS, particularly regarding career opportunities, specialised skills and the nature of the specialty. However, their awareness of the full scope of plastic surgery is limited, with a focus on cosmetic and aesthetic procedures. Social media and the internet significantly influenced the students' perceptions, whereas personal experiences had a minor impact. Education and training in plastic surgery positively affected the students' perceptions. Nevertheless, there is a need for improved representation of PRAS in medical school curricula and promotion of accurate information through reliable sources.

**Conclusion:**

Students exhibited a favourable attitude towards plastic surgery, but their knowledge of the specialty can be enhanced. Strengthening PRAS teaching in medical schools and ensuring accurate information dissemination can foster a deeper understanding and interest in this field. Large-scale studies with standardised protocols should be conducted in different countries to gain comprehensive insights tailored to specific educational contexts.

## Introduction

Plastic, reconstructive and aesthetic surgery (PRAS) is an underrepresented surgical specialty in medical school curricula.^1^^,^^2^ Being well-informed helps future practitioners make informed decisions regarding their career paths, thus reducing issues related to professional satisfaction, burnout and work-life balance. Additionally, medical students, as future healthcare providers, play a crucial role in shaping public perceptions about plastic surgery. They also contribute to making suitable and appropriate referral, thereby reducing the burden on the patients and healthcare services.^3^^,^^4^

In this era of rapid technological changes, the profile of medical students constantly evolves. Perceptions and awareness of medical specialties are significantly influenced by the widespread use of social media and unmoderated online sources, leading to misconceptions about a career in plastic surgery.^5^ At present, no high-quality information is available and career advice is provided to medical students by professional organisations such as the British Association of Plastic, Reconstructive and Aesthetic Surgeons (BAPRAS). Several UK and internationally based studies have attempted to understand the attitudes of medical students towards a career in plastic surgery; nonetheless, the influences and awareness are yet uncertain. For professional bodies to efficiently disseminate accurate information, there is a need of primarily understanding the current landscape.

This systematic review had the following aims:

(i) to determine the positive and negative perceptions about PRAS among medical students
(ii) to identify the factors influencing these perceptions
(iii) to examine the existing strategies for addressing these influences.

## Methods

A systematic literature review was performed in accordance with the 2020 preferred reporting items for systematic reviews and meta-analyses (PRISMA) guidelines.^6^ The literature search was conducted in December 2022. A comprehensive search of published, peer-reviewed scientific literature was performed using Pubmed, EMBASE, Web of Science and PsycInfo databases. The search string included the following keywords: ‘medical student’, ‘medical students’, ‘student doctors’, ‘med students’, ‘perception’, ‘viewpoint’, ‘determinant’, ‘factors impacting’, ‘point of view’, ‘influence’, ‘awareness’, ‘plastic surgery’, ‘plastics’, ‘aesthetic surgery’ and ‘reconstructive surgery’. The selection was confined to studies published in the past decade, reflecting the growing influence of social media and evolving characteristics of contemporary medical students.

Subjective perceptions of medical students about plastic surgery were evaluated and strategies for overcoming the misconceptions were addressed. The awareness of procedures within the purview of a plastic surgeon was assessed to identify the gaps in knowledge of medical students and to help direct education strategies. Finally, the career-choice influencing factors were explored.

### Selection and data collection process

Studies were included for further assessment if they met the inclusion criteria listed in Table 1.

**Table 1:** Inclusion and exclusion criteria for the studies used in this systematic review.

Inclusion	Exclusion
(1) Written in English	(1) Letters to editors, editorials, systematic reviews, literature reviews, expert opinions, meta-analysis and grey literature
(2) Full free text available	(2) Reported data from healthcare professionals, surgical trainees/residents and members of the general public
(3) Reported data from medical students in years 1-6 including intercalating students	(3) Data on surgery in a general sense with no specific focus on plastic surgery
(4) Data focused on plastic surgery	(4) Studies with data on the exposure of medical students to surgery in a general sense
(5) Studies with data on exposure of medical students to plastic surgery	
(6) Empirical observational studies including cohort studies, case-control studies and randomised control trials	

All electronic search results were uploaded on COVIDENCE,^7^ a primary screening and data extraction tool for conducting standard intervention reviews. Upon initial search and duplicate removal, two independent reviewers (SPG, CAP) screened all the titles and abstracts for relevant studies, using the defined inclusion and exclusion criteria. All remaining articles underwent independent full-text review and were evaluated for eligibility (using the inclusion criteria) and relevance (using the described outcomes) by the same reviewers. Data were manually extracted in standardised spreadsheets using Microsoft Excel. Three independent reviewers (AG, SPG and CAP) discussed and addressed data discrepancies.

### Data items

Initial data extraction included a collection of the following information from all studies: authors, publication year, type of study, country of origin and participant demographics such as sex, age and year of study (pre-clinical or clinical). An exhaustive extraction of data was conducted separately for the three divisions of the primary objective of the systematic review, namely perceptions, awareness and influences.

### Synthesis methods

The analysis was divided into three categories: perceptions, awareness and influences. For the ‘Perceptions’ analysis, all studies that provided insight into the perceptions of medical students regarding the plastic surgery specialty, the profession itself, its inherent nature, and the training associated with it were included.

Awareness category focused on surgical procedures performed by plastic surgeons or those related to the specialty were selected. Influences category comprised studies on information sources and factors affecting the perceptions and awareness of the students.

All aspects of perception/awareness/influence were considered, categorically organised for clarity and assessed by three reviewers (AG, SPG and CAP). Perceptions were classified as positive, negative or mixed, with 55 distinct perceptions qualitatively coded into 20 subthemes. For awareness and influences, data were grouped and coded based on its nature. Mean percentages were calculated for each category across studies. In cases where data clarity was an issue, increments of 10.0 % were used. Outcomes were represented in stacked bar charts, with secondary outcomes showing percentage changes in pre- and post-course responses or between different knowledge levels, along with mean and range values.

### Study risk of bias assessment

To assess the risk of bias in these studies, an adapted version of the Newcastle–Ottawa Scale (NOS) for cohort and case-control studies was used^8^ (Appendix 1). For cohort studies, the NOS tailored for cohort studies was used. For randomised controlled trials, the NOS for cross-sectional studies were used.

Two independent reviewers (AG and SPG) entered the data into Microsoft Excel. Any discrepancies or disagreements were resolved through discussion among the reviewers. The independent evaluation scores can be found in Appendix 2. The quality of studies was rated as ‘good’ (≥6 points), ‘fair’ (2-5 points) or ‘poor’ (≤1 point).

## Results

### Study selection

**Figure 1:**
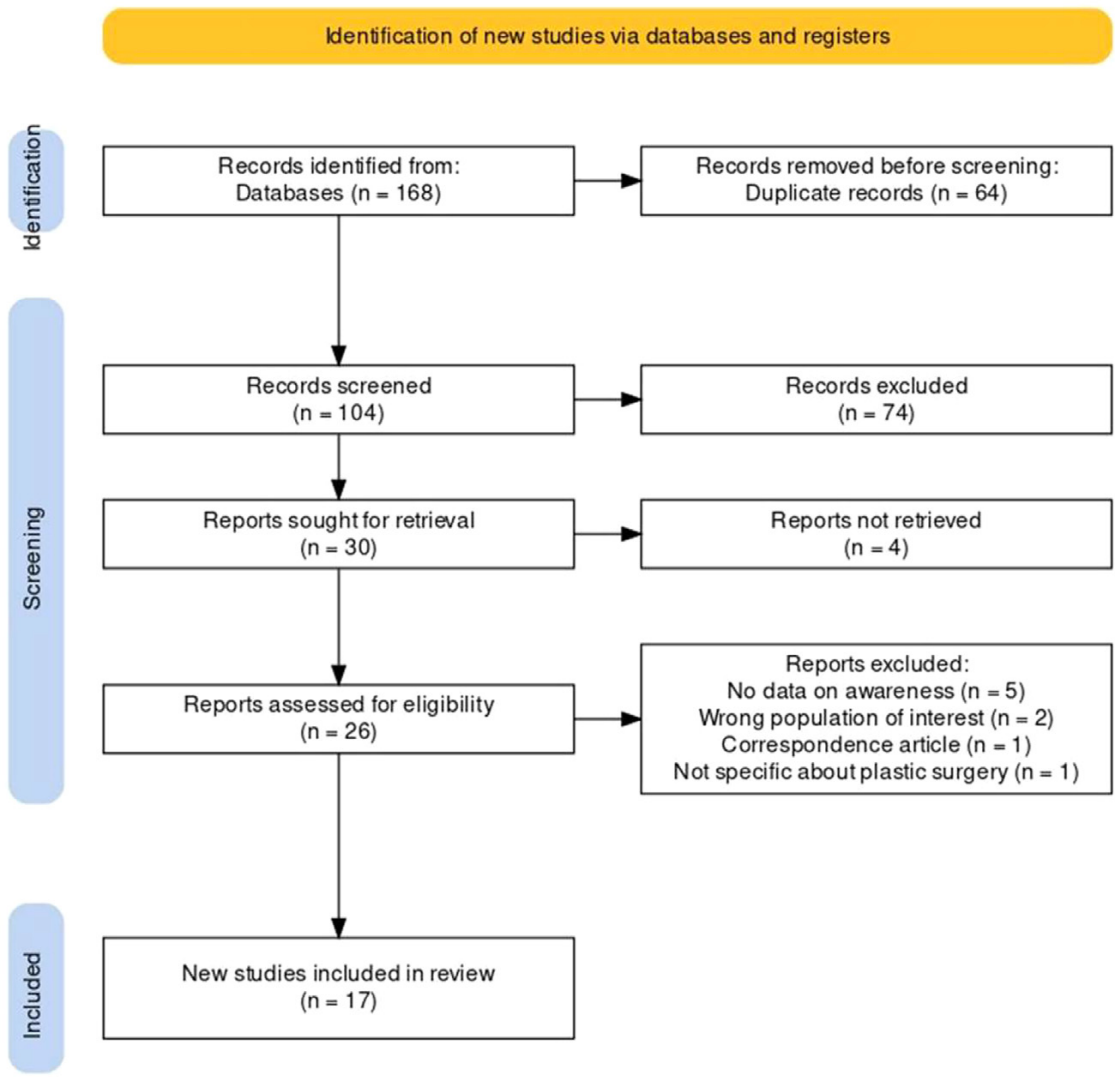
Flow diagram representation of study inclusion generated via PRISMA 2020: An R package and Shiny app were used for producing PRISMA 2020-compliant flow diagrams, with interactivity for optimised digital transparency and Open Synthesis Campbell Systematic Reviews.^42^

The database search initially retrieved 168 studies with 64 duplicates removed, and 74 studies further excluded based on the inclusion and exclusion criteria. Thirty papers were subjected to full-text review, with 17 being included in the review. Figure 1 illustrates the flowchart of the literature search and inclusion process.

The results of the quality and risk of bias of studies can be viewed in Appendix 2. Notably, no primary studies in this review scored less than 1 point overall, for their quality to be classified as ‘poor’. Sixteen studies scored as ‘fair’ and one as ‘good’.

### Study characteristics

The studies encompassed 6,142 participants, with a mean age of 22.9 years. Gender was reported in 12 studies totalling 5,770 participants (2,880 women and 2,890 men). Thirteen studies included in the review, reported the academic year of the participants, with 41% being in their pre-clinical phase and 59% in their clinical phase as classified by the standards of each respective country. Data on gender and academic year were not provided in 5 and 4 studies, respectively (Table 2).

**Table 2:** Summary of articles that met the inclusion criteria and their participant demographics.

Table 1: Studies included and participant demographics									
Study	Study type (survey, course)	Year of publication	Country	Number of students (n)	Sex (n, %)		Mean age (years)	Year (n,%)	
					Females	Males		Preclinical	Clinical
**Khatib et al.**^18^	Survey pre- and post-course	2015	United Kingdom	39	Not recorded	Not recorded	Not recorded	Not recorded	Not recorded
**Davis et al.**^12^	Survey pre- and post-course	2016	United Kingdom	175	99 (56.6)	74 (42.3)	Not recorded	23 (13.1)	152 (86.9)
**Singh et al.**^21^	Survey pre- and post-course	2022	United States	168	Not recorded	Not recorded	Not recorded	153 (91.1)	14 (8.9)
**Almeland et al.**^9^	Survey pre- and post-course	2020	Norway	45	Not recorded	Not recorded	Not recorded	45 (100)	0 (0)
**Spiers et al.**^22^	Survey, pre- and post-course	2018	United Kingdom	23	Not recorded	Not recorded	Not recorded	5 (22)	18 (78)
**Alyahya et al.**^10^	Survey, no course	2021	Saudi Arabia	292	103 (35.3)	189 (64.7)	Not recorded	174 (59.6)	118 (40.4)
**Austin and Wanzel**^11^	Survey, no course	2015	Canada	354	217 (61.3)	135 (38.1)	25	Not recorded	Not recorded
**Conyard et al.**^13^	Survey, no course	2016	Australia	234	141 (60.3)	93 (39.7)	Not recorded	115 (49.1)	119 (50.9)
**Farid et al.**^2^	Survey, no course	2017	Canada and United Kingdom	243	125 (51.4)	118 (48.6)	Not recorded	Not recorded	Not recorded
**Fayi et al.**^14^	Survey, no course	2018	Saudi Arabia	441	0 (0)	441 (100)	22.3	64 (14.5)	377 (85.5)
**Fraser et al.**^15^	Survey, no course	2017	Canada	214	124 (58)	90 (42)	Note recorded	Not recorded	Not recorded
**Gathariki et al.**^16^	Survey, no course	2020	Kenya	108	60 (55.6)	48 (44.4)	Not recorded	28 (26)	80 (74)
**Jabaiti et al.**^23^	Survey, no course	2021	Jordan	200	107 (53.5)	93 (46.5)	23.1	0 (0)	200 (100)
**Kidd et al.**^3^	Survey, no course	2021	United Kingdom	194	132 (67.9)	61 (31.6)	Not recorded	92 (47.4)	101 (52.1)
**Kling et al.**^19^	Survey, no course	2014	United States	2434	1329 (54.6)	1105 (45.4)	Not recorded	1222 (50.2)	1212 (49.8)
**Mehta et al.**^20^	Survey, no course	2016	India	92	Not recorded	Not recorded	Not recorded	92 (100)	0 (0)
**Mortada et al.**^17^	Survey, no course	2019	Saudi Arabia	886	443 (50)	443 (50)	21	156 (17.6)	729 (82.3)
**Total**				6,142	2,880	2,890	22.9 (mean)	2,169 (41)	3,120 (59)

### Perception results

Perceptions were divided into positive, negative and mixed with 19, 24 and 12 unique perceptions, respectively. Among the selected studies, the specific perceptions included in each category, along with the frequency of occurrence across the selected studies, can be found in Table 3, Table 4, Table 5.

**Table 3:** Grouping of positive perceptions into larger subcategories.

Positive perceptions		
Category	Perceptions included	Included in (n) studies
**Nature of specialty**	Challenging (2), Creative (2), Artistic (1), Rewarding (1), Rapidly growing (1), Life changing (1) and Interesting (1)	9
**Lifestyle**	Lifestyle (2) and Income (5)	7
**Opportunities**	Research (3), Travel (1), Management (1), Teaching (1), Private practice (2) and Voluntary work (1)	9
**Patient Interaction**	Patient Interaction (2)	2
**Operative satisfaction**	Operative satisfaction (1)	1
**Skill requirement**	Skill requirement (4)	4
**Positive Outlook**	Positive Outlook (1)	1
**Total positive perceptions reported (n):** 33		
**Total unique positive perceptions reported (n):** 19		
**Studies reporting positive perceptions (n):** 9		

Positive perceptions were reported in 9 studies, and these were categorised as: nature of specialty, lifestyle, opportunities, patient interaction, operative satisfaction, skill requirement and positive outlook.

**Table 4:** Grouping of negative perceptions into larger subcategories.

Negative perceptions		
Category	Perceptions included	Included in (n) studies
**Nature of the specialty**	Boring/lack of interest (3), Superficial (2), Delicate and risky (3), Negative implications of cosmetic surgery (1) and Stressful (1)	10
**Value of procedures**	Non-life saving (4), Luxurious (2) and Not proper medicine (1)	7
**Nature of training**	Long training (3) and Demanding (1)	4
**Specialty competitiveness**	Job availability (1) and Difficulty entering residency (2)	3
**Ethical doubts**	Unethical (2) and Religious concerns (2)	4
**High chance of litigation**	High chance of litigation (2)	2
**Opinions of others about the specialty**	Discouragement from mentors and peers (1), Social stigma (2), Negative stereotypes of surgeons (1), Disliked by other specialties (1), Difficult social acceptance (2), Negative public perception (1), Negative outlook (1) and Personality (1)	10
**Total negative perceptions reported (n):** 40		
**Total unique negative perceptions reported (n):** 24		
**Studies reporting negative perceptions (n):** 11		

Negative perceptions were reported in 11 studies and were categorised as nature of the specialty, value of procedures, nature of training, specialty competitiveness, ethical and personal doubts, high chance of litigation and opinions of others about the specialty.

**Table 5:** Grouping of mixed perceptions into larger subcategories.

Mixed perceptions		
Category	Perceptions included	Included in (n) studies
**Nature of specialty**	Hardworking (1) and Competitive (3)	4
**Procedure variety**	Procedure variety (4), Variety (1), Multi-system/multi-organ (2) and Diverse (2)	9
**Focus on cosmetic aspects**	Focus on cosmetic aspects (6)	6
**Impact on patients**	Difference in people's lives (3) and Improve quality of life of the patient (3)	6
**Work schedule**	Long working hours (3) and On-call duty (2)	5
**Working with other specialties**	Working with other specialties (3)	3
**Total mixed perceptions reported (n):** 33		
**Total unique mixed perceptions reported (n):** 12		
**Studies reporting mixed perceptions (n):** 8		

Mixed perceptions were reported in 8 studies and were categorised as nature of specialty, procedure variety, focus on cosmetic aspect, impact on patients, work schedule and working with other specialties.

**Figure 2:**
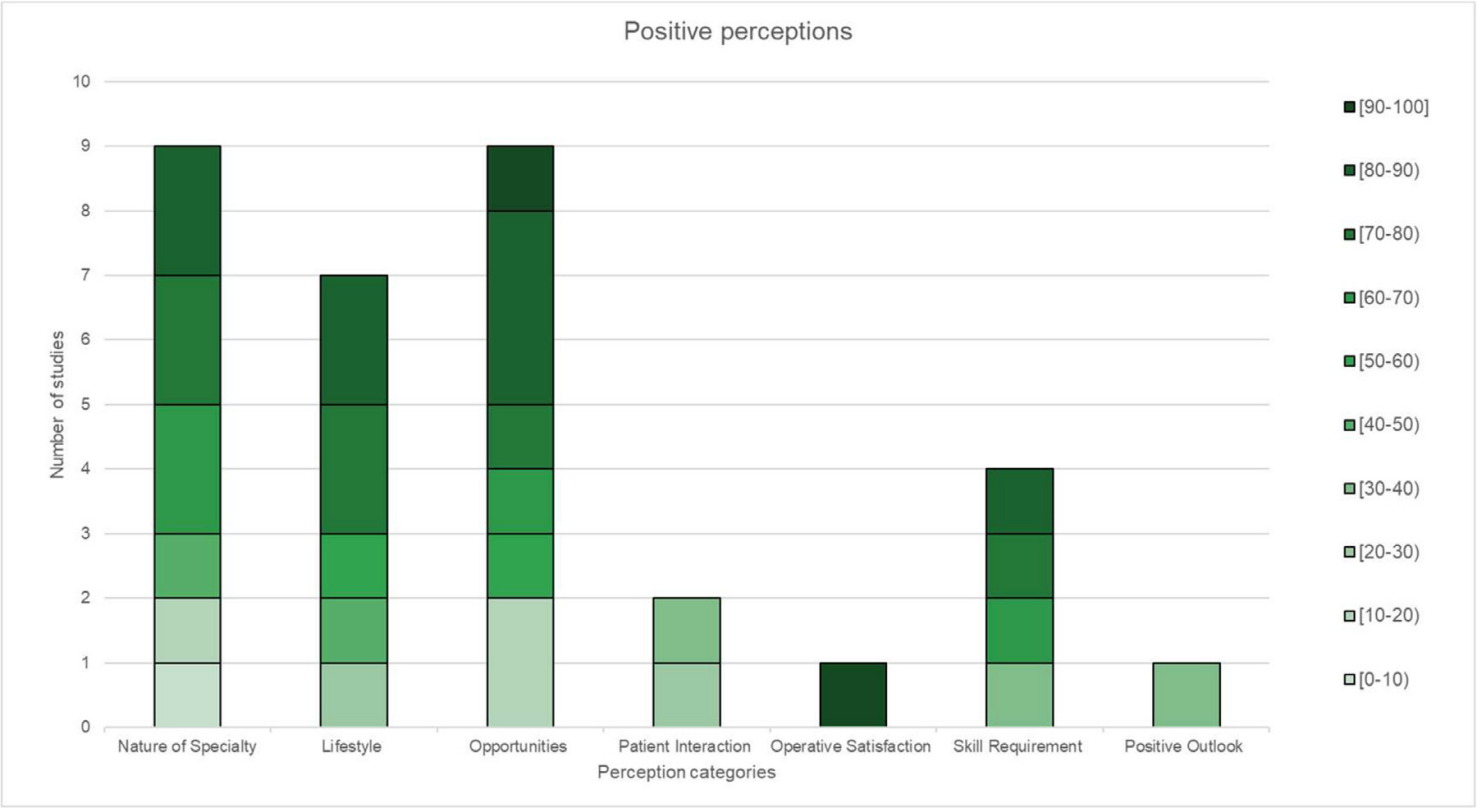
Distribution of positive perceptions presented in 10.0% increments.

**Figure 3:**
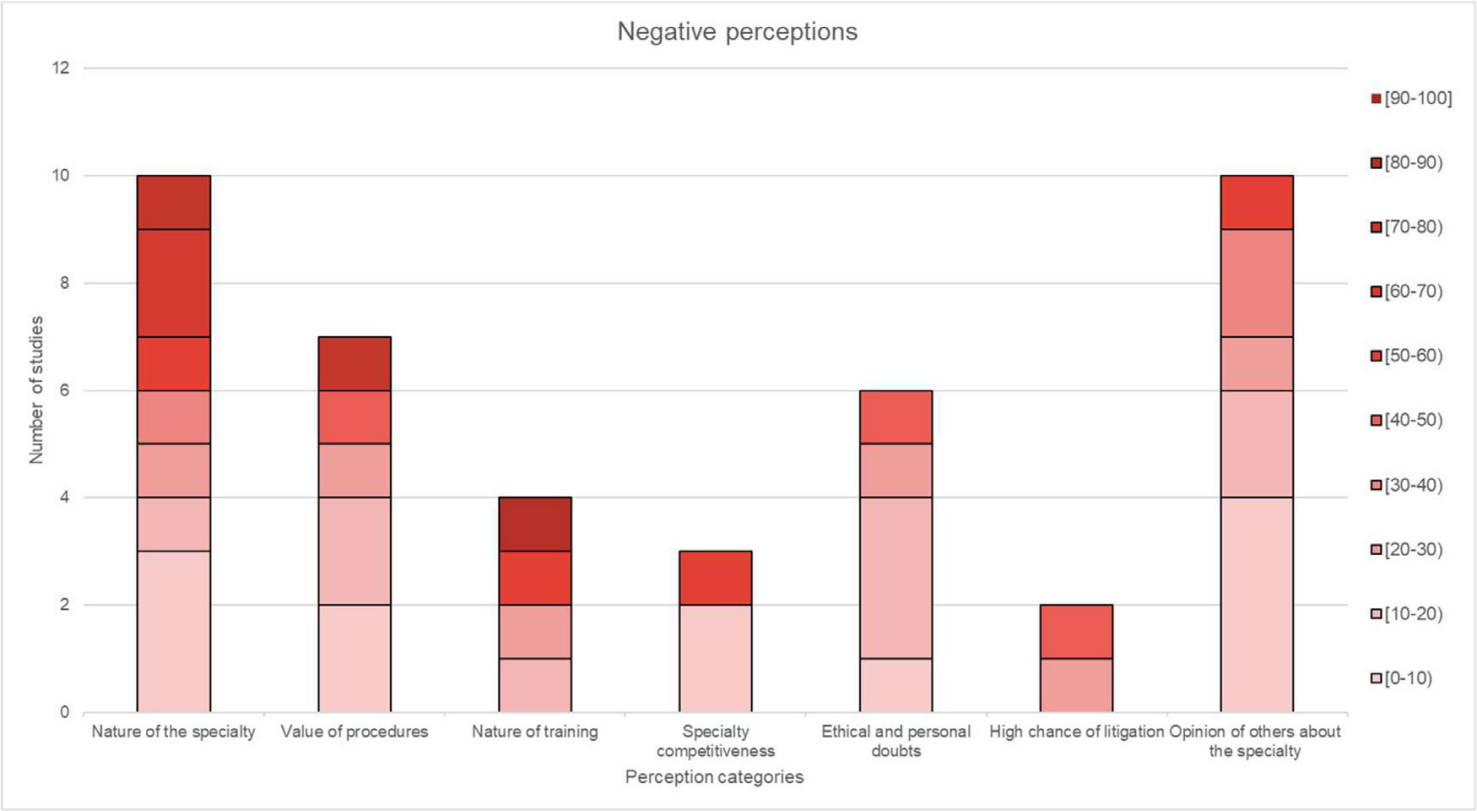
Distribution of negative perceptions in 10.0% increments.

**Figure 4:**
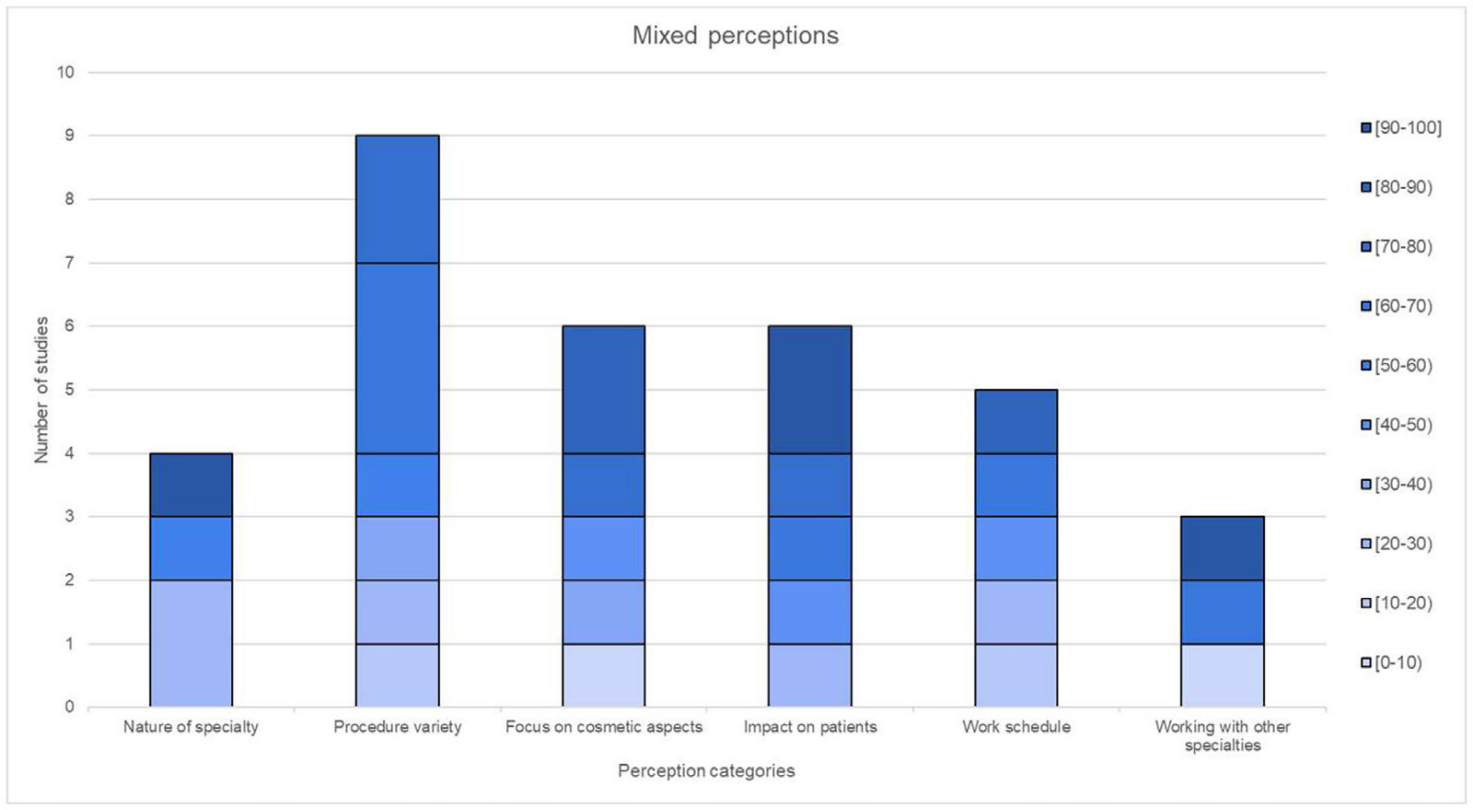
Distribution of mixed perceptions in 10.0% increments.

Perceptions were also analysed based on the percentage value representing the agreement of the study participants with each perception, classified in 10.0% increments. The distribution of the positive, negative, and mixed perceptions can be seen in Figure 2, Figure 3, Figure 4, respectively. For the positive perceptions, the average agreement rate varied considerably, for example, patient interaction had an average value of 30.0% whereas operative satisfaction was 95.0% (Figure 2). For negative perceptions, the average value was less than 50.0% for all perceptions, with ethical doubts having the lowest average at 20.0% (Figure 3). Mixed perceptions displayed had an average value of approximately 50.0%, as expected, because these perceptions were viewed as positive by some respondents and negative by others. For instance, responses concerning the ‘Impact on patients’ and ‘Nature of the specialty’ had mean values of 66.7% and 50.0%, respectively (Figure 4).

### Awareness results

To measure the awareness of medical students regarding plastic surgery, 21 categories of procedures were identified. Notably, from the studies that included a course, the initial analysis of results involved extracting data on the students' pre-course knowledge. Commonly recognised procedures included cosmetic and aesthetic surgery, breast reconstruction, burns and gender reassignment. Conversely, procedures such as hand surgery/trauma, head and neck cancer, pressure ulcers, arthritis and tendon surgeries were the least recognised.

**Figure 5:**
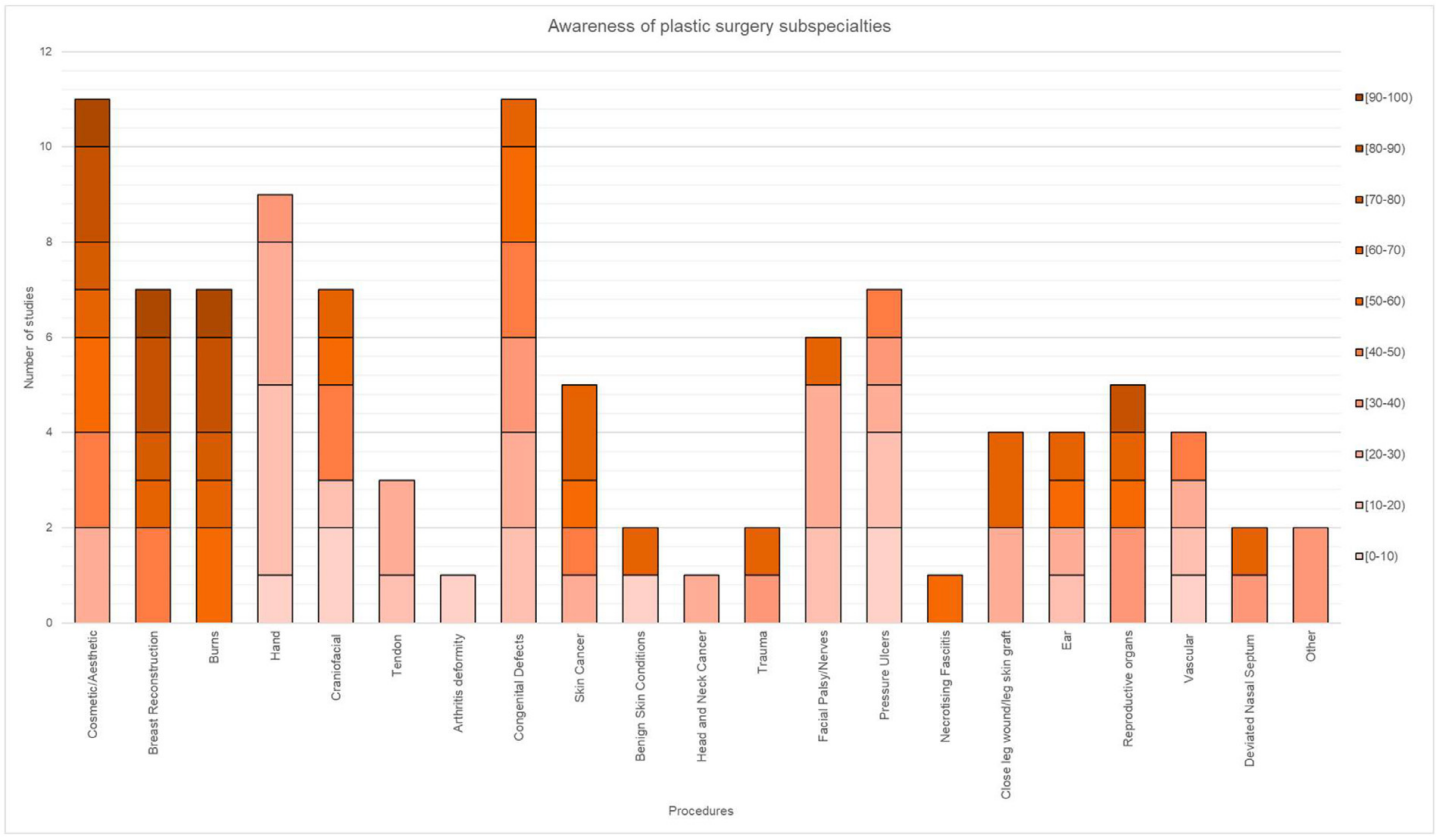
Distribution of awareness of procedures in 10.0% increments.

Despite hand trauma constituting over 50.0% of the workload performed by plastic surgeons, the reported average awareness for hand/trauma surgery was only 19.4% (Figure 5).^24^ In contrast, cosmetic/aesthetic procedures saw a higher awareness, with 7 out of 11 studies showing over 50.0% awareness, averaging at 59.5%.

### Influences

**Figure 6:**
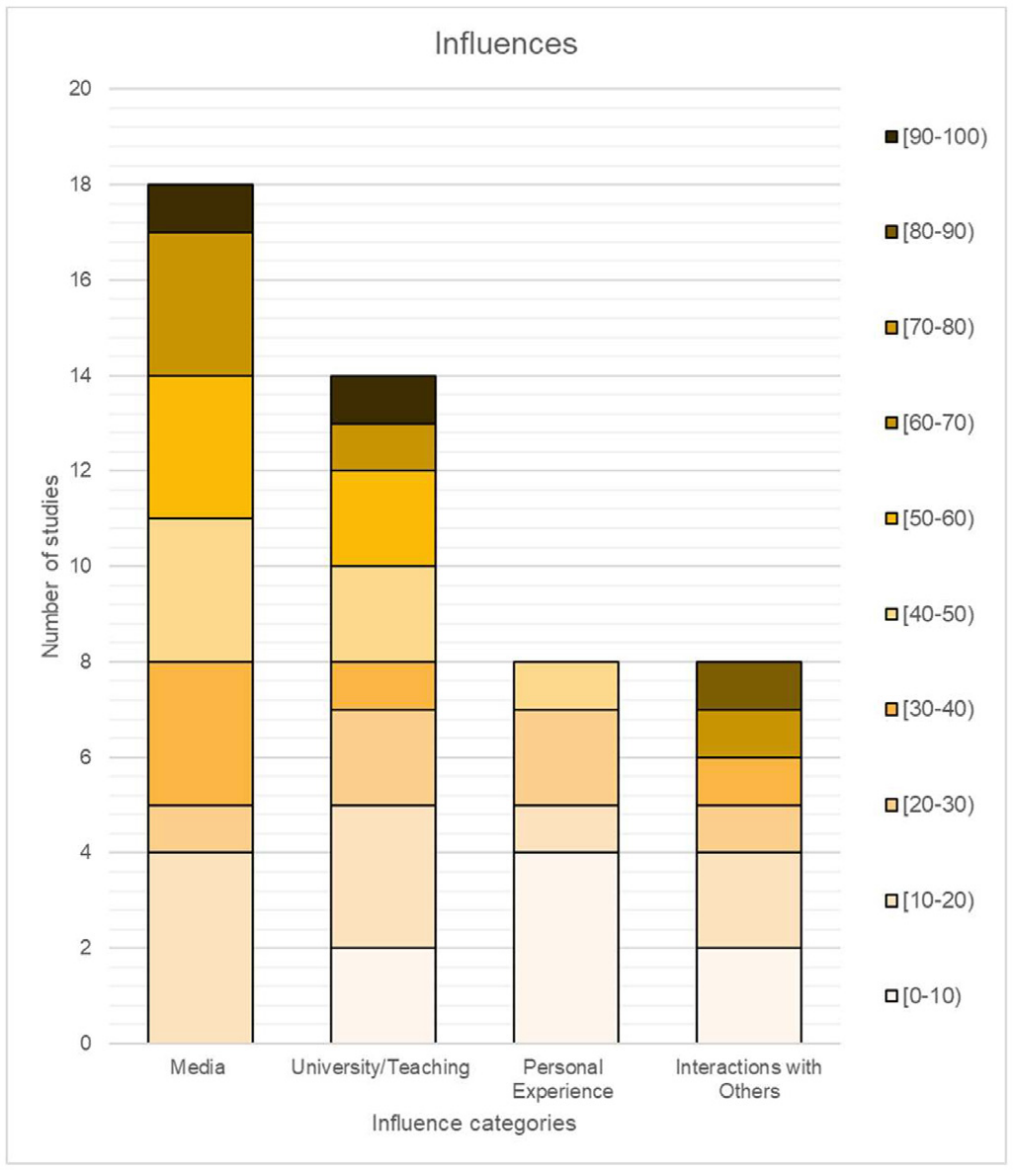
Distribution of influences presented in 10.0% increments.

Influences were categorised into four groups: media, university/teaching, personal exposure and interactions with others. Across the included studies, a total of 48 influences were reported, encompassing 15 distinct types. The category of ‘Media’ was the most frequently mentioned influence, with a total of 18 mentions in the 12 studies that reported influences. However, the influence of ‘Personal experience’ and ‘Interaction with others’ were the least selected, with only eight mentions in the same number of studies (Figure 6).

## Discussion

### Perceptions

The results for positive perceptions predominantly showed high percentages, often exceeding 50.0%. This indicates that most students viewed aspects such as favourable lifestyle, diverse career opportunities and specialised skills positively in the realm of plastic surgery. Additionally, they perceived the nature of the specialty as a positive characteristic, although only 30.0% of students identified ‘patient interaction’ as a positive impact. Overall, these findings demonstrate a general positive outlook on plastic surgery in terms of the lifestyle and skills it incorporates.

Conversely, only a small percentage of respondents viewed aspects such as ‘values of the procedures’, ‘specialty competitiveness’ and ‘ethical and personal doubts’ negatively. These results align with other studies indicating the public's perception of plastic surgery as less critical in delivering patient care.^25^

For some perceptions, the data did not follow a specific trend: three were negative (nature of specialty, nature of training and high chance of litigation) and four were mixed (procedure variety, impact on patients, work schedule and working with other specialties). No definitive conclusions could be drawn about the positive ‘operative satisfaction’, ‘positive outlook’ or negative ‘high chance of litigation’ given a low number of studies reporting on these perceptions.

These findings show a need for dissemination of accurate information regarding a career in PRAS. Professional bodies such as BAPRAS can address the underrepresentation of plastic surgery in medical education.^26^ This can be done by providing a balanced portrayal that includes the challenges and rewards of the field, such as verified social media posts and internet resources.^27^ Additionally, adding guest lectures and seminars in the medical curriculum will offer students with insights into the advancements in the field, potentially dispelling myths about the profession.^22^ Lastly, collaborative initiatives in PRAS research and shadowing opportunities can help solidify their understanding on the importance of plastics for patient care.^28^

### Awareness

In the studies examining awareness about PRAS among medical students, variations in their interest in pursuing a career in this field make it difficult to establish a clear link between self-reported interest and awareness of PRAS. However, the study by Kling et al. showed that students who were already interested in plastic surgery were better at correctly identifying the subspecialties of PRAS.^19^ Other research papers have highlighted a desire among students for more PRAS education and clinical rotations, suggesting an eagerness to enhance their understanding of the field. This further demonstrates the lack of PRAS teaching and high demand for further exposure among the students. Yet, the proportion of medical schools in the United Kingdom including plastic surgery teaching in the medical undergraduate curriculum has decreased to 13.0% in 2008, with several aspects of plastic surgery falling under the curricula of other surgical specialties.^29^ Additionally, according to Au and Kim, only two medical schools in the UK have compulsory PRAS teaching,^30^ whereas Austin and Wanzel revealed a mere of 2 h of plastic surgery teaching, without mandatory PRAS rotation in Canada.^11^

Considering that 89.0% percent of articles published in the press in the United Kingdom referred to PRAS in the context of cosmetic surgery and only 10.0% addressing its reconstructive aspect,^31^ it is important to consider the external influences on students’ awareness of plastic surgery, as they could supersede their educational ones. Overall, our findings suggest that medical students have a limited understanding of the breadth of subspecialties in plastic surgery. Although cosmetic and aesthetic procedures were more correctly attributed to the specialty, there was little appreciation of the full scope of plastic surgery and its daily operations. For those choosing not to pursue a career in PRAS, this limited understanding and appreciation of the scope of the specialty might impede their ability to make appropriate specialist referrals.^32^

### Influences

The analysis of the results revealed that the least chosen influence was personal experience. This, coupled with the underrepresentation of plastic surgery in medical schools leads medical students to rely on alternative sources for information. Naturally, as medical students progress through their clerkship in the medical school, their knowledge and perceptions regarding medical and surgical specialties should increase due to university-based instructions.^33^ Studies comparing pre-clinical years^9^^,^^21^ and clinical years,^12^^,^^16^^,^^17^^,^^23^ found media to be a primary source of information. Ultimately, the lack of foundational knowledge in plastic surgery adversely impacted students who gravitate towards a career in plastic surgery and hindered their progression in this competitive field.^17^ The study suggests directing students towards credible resources via their medical education to mitigate misinformation risks and poor clinical practice stemming from invalid sources of information.^34^

Media, including TV, social media and internet search, were identified as the most influencing factor and primary source of information. This contrasts with the studies prior to 2010 which suggested that lectures and student-surgeon interactions were the primary influence on students’ perceptions and awareness of plastic surgery.^35^^,^^36^ The evolution of a readily available diverse group of media platforms and internet networking may alter the attitudes towards and awareness of the specialty that may or may not be accurate. Another example is YouTube, a widely used platform, where the quality of educational information has been evaluated as being poor.^37^ Given the inevitable rise of social media, particularly amongst the new generation of medical professionals, promoting a more truthful and realistic portrayal of medicine should be strongly encouraged and promoted.^38^ Delivering high-quality online sources of information that match the style of learning among the current students has become increasingly important. As social media is a powerful tool for the dissemination of accurate, engaging and educational content to a wider audience, it needs to be recognised and utilised to help the specialty.^39^

### Secondary outcome

The secondary outcome arising from the review evaluated the impact of courses and/or educational workshops on the awareness and perceptions about plastic surgery, aiding in the development of appropriate interventions.

Among the included studies, 5 used a course or workshop to compare pre- and post-intervention levels of awareness. The effectiveness of these educational interventions was analysed by observing changes in the positive, negative and mixed perceptions, totalling 40 variations. Notably, five specific negative perceptions decreased, and two positive perceptions increased. Notably, the perception ‘focus on cosmetic aspect’ responses decreased by 65.0% whereas perceptions related to ‘work schedule’, ‘nature of specialty’ and ‘impact on patients’ increased by a mean of 40.0%, 22.0% and 16.0%, respectively. This indicates that education or training has the potential to shift the student's perceptions about the specialty. Another study suggested that this can be extended to procedural awareness before an educational programme, as 85.0% of final-year medical students could not list more than five conditions treated by plastic surgeons; this figure reduced to zero post-programme.^30^ This is supported by a pre-2010 study which indicated that exposure to plastic surgery, specifically when courses on topics such as cleft surgery were introduced, improved awareness of the specialty's scope.^40^ Overall, standardising medical school curricula to include PRAS rotations and diverse teaching modalities may provide a better view of the specialty.

## Limitations of evidence used in the review

This systematic review is subject to inherent limitations and a certain level of bias. Among the papers selected for analysis, 15 were observational cross-sectional studies, 1 was an observational cohort study, and only one was a randomised controlled trial; most of which fall towards the lower end of the evidence hierarchy.^41^

Moreover, there was an uneven representation of countries in this study despite its global scope. Consequently, the evidence was predominantly focused on the United States, Canada, United Kingdom and Saudi Arabia, with limited representation from other nations. As many of these papers were produced by medical students themselves, this is potentially due to the demands of training applications or more opportunities available for research. Simultaneously, the variations in medical curricula across different countries and absence of standardised protocols for questionnaires further contribute to the heterogeneity of the evidence. Additionally, several study questionnaires were not piloted before their launch or piloting was not reported. Lastly, certain studies included only positive perceptions in their questionnaires, leading to a bias favouring a positive attitude towards plastic surgery.

## Limitations in the review process used

Considering the absence of precise quantitative data in certain studies, where results were presented in the form of figures without explicit numerical values, it became necessary to extrapolate the data in increments of 10.0%. This approach was employed to derive approximate values for the purpose of analysis.

It is important to acknowledge the potential introduction of unconscious researcher bias during the design and implementation of the study, which may have influenced the study's direction towards the anticipated outcome.

## Conclusion

Overall, medical students have a favourable attitude towards plastic surgery. However, students are most aware of cosmetic, reconstructive and burn management aspects of PRAS, with limited knowledge concerning other subspecialties within the field. Owing to the ever-increasing popularity of social media and the internet platforms, it is crucial to promote the dissemination of accurate information regarding plastic surgery. Moreover, there is a strong recommendation to enhance the curricular exposure and teaching of plastic surgery within medical schools. Such measures have the potential to benefit the students and medical profession as a whole, by fostering a deeper understanding and proficiency in this area. Lastly, it is advisable to conduct an extensive review of the medical curricula in different countries. Specific educational contexts, teaching requirements and the needs of different areas should be considered to adapt teaching opportunities to achieve better awareness and perceptions of medical students regarding PRAS.

## Declaration of competing interest

The authors declare there is no conflict of interests.

## Appendix Supplementary materials

Image, application 1
