# Supplementary material for: Perceptions, awareness and influences of medical students towards plastic surgery: A systematic review
Source: JPRAS Open. 2024 Apr 8;40:320–35. doi: 10.1016/j.jpra.2024.04.003 (PMC11081776; doi:10.1016/j.jpra.2024.04.003)
Supplement: Supplementary file 1 [file mmc1.docx]

APPENDIX/Supplementary material

**Appendix 1**

NOS Adapted cross sectional

Selection:

Representativeness of the sample:

Truly representative of the average in the target population. * (all subjects or random sampling)

Somewhat representative of the average in the target group. * (non-random sampling)

Selected group of users/convenience sample.

No description of the derivation of the included subjects.

Sample size:

Justified and satisfactory (including sample size calculation). *

Not justified.

No information provided

Non-respondents:

Proportion of target sample recruited attains pre-specified target or basic summary of non-respondent characteristics in sampling frame recorded. *

Unsatisfactory recruitment rate, no summary data on non-respondents.

No information provided

Ascertainment of the exposure (risk factor):

Vaccine records/vaccine registry/clinic registers/hospital records only. **

Parental or personal recall and vaccine/hospital records. *

Parental/personal recall only.

Comparability: (Maximum 2 stars)

Comparability of subjects in different outcome groups on the basis of design or analysis. Confounding factors controlled.

Data/ results adjusted for relevant predictors/risk factors/confounders e.g. age, sex, time since vaccination, etc. **

Data/results not adjusted for all relevant confounders/risk factors/information not provided.

Outcome:

Assessment of outcome:

Independent blind assessment using objective validated laboratory methods. **

Unblinded assessment using objective validated laboratory methods. **

Used non-standard or non-validated laboratory methods with gold standard. *

No description/non-standard laboratory methods used.

Statistical test:

Statistical test used to analyse the data clearly described, appropriate and measures of association presented including confidence intervals and probability level (p value). *

Statistical test not appropriate, not described or incomplete.

Cross-sectional Studies:

Very Good Studies: 9-10 points

Good Studies: 7-8 points

Satisfactory Studies: 5-6 points

Unsatisfactory Studies: 0 to 4 points

This scale has been adapted from the Newcastle-Ottawa Quality Assessment Scale for cohort studies to provide quality assessment of cross sectional studies.

Herzog R, et al. Is Healthcare Workers’ Intention to Vaccinate Related to their Knowledge, Beliefs and Attitudes? A Systematic Review. BMC Public Health 2013 13:154

**Appendix 2**

Risk of bias results

| **Domain** | **Selection (4 points maximum)** | | **Comparability (1 point maximum)** | | **Outcome (2 points maximum)** | | **Total Score (7 points maximum)** | | **Judgement** | |
| --- | --- | --- | --- | --- | --- | --- | --- | --- | --- | --- |
| Reviewer | AG | CG | AG | CG | AG | CG | AG | CG | AG | CG |
| [**Austin and Wanzel**](https://www.pulsus.com/scholarly-articles/exposure-to-plastic-surgery-during-undergraduatemedical-training-a-singleinstitution-review.pdf) | 2 | 2 | 0 | 0 | 1 | 2 | 3 | 4 | Fair quality | Fair quality |
| [**Fraser et al**](https://journals.sagepub.com/doi/epub/10.1177/2292550317694844) | 2 | 2 | 0 | 0 | 1 | 1 | 3 | 3 | Fair quality | Fair quality |
| [**Jabaiti et al**](https://journals.sagepub.com/doi/10.4081/jphr.2021.1927) | 4 | 3 | 1 | 1 | 2 | 2 | 7 | 6 | Good quality | Good quality |
| [**Kling et al**](https://journals.lww.com/plasreconsurg/Abstract/2014/04000/The_Scope_of_Plastic_Surgery_According_to_2434.33.aspx) | 2 | 2 | 1 | 1 | 2 | 2 | 5 | 5 | Fair quality | Fair quality |
| [**Mehta et al**](https://www.jemds.com/data_pdf/Pawan.pdf) | 0 | 0 | 1 | 1 | 1 | 1 | 2 | 2 | Fair quality | Fair quality |
| [**Spiers et al**](https://www.sciencedirect.com/science/article/abs/pii/S1931720417302726?via%3Dihub) | 2 | 0 | 1 | 0 | 2 | 2 | 5 | 2 | Fair quality | Fair quality |
| [**Alyahya et al**](https://journals.sagepub.com/doi/epub/10.1177/20503121211054373) | 0 | 0 | 1 | 1 | 2 | 2 | 3 | 3 | Fair quality | Fair quality |
| [**Conyard et al**](https://www.sciencedirect.com/science/article/pii/S2352587816000036) | 3 | 1 | 1 | 1 | 2 | 2 | 6 | 4 | Good quality | Fair quality |
| [**Davis et al**](https://www.sciencedirect.com/science/article/abs/pii/S174868151630064X) | 2 | 2 | 1 | 1 | 2 | 2 | 5 | 5 | Fair quality | Fair quality |
| **Farid et al** | 2 | 2 | 1 | 0 | 0 | 0 | 3 | 2 | Fair quality | Fair quality |
| [**Kidd et al**](https://reader.elsevier.com/reader/sd/pii/S2352587821000401?token=AB9751BED4B5A71FA588E6B35A3FBDC6DEBF3521BAC14CA2FAA517BFDF593F15942A082E8A613F4EBB141DBDEC8EAEE6&originRegion=eu-west-1&originCreation=20230212143754) | 2 | 2 | 1 | 1 | 1 | 1 | 4 | 4 | Fair quality | Fair quality |
| [**Mortada et al**](https://www.i-jmr.org/2019/2/e12999/) | 2 | 1 | 1 | 1 | 2 | 2 | 5 | 4 | Fair quality | Fair quality |
| [**Fayi et al**](https://journals.lww.com/jfmpc/Fulltext/2018/07060/Male_medical_students__perception_of_plastic.59.aspx) | 1 | 1 | 1 | 1 | 2 | 2 | 4 | 4 | Fair quality | Fair quality |
| [**Almeland et al**](https://link.springer.com/content/pdf/10.1007/s00238-019-01615-w.pdf?pdf=button) | 2 | 2 | 1 | 0 | 2 | 2 | 5 | 4 | Fair quality | Fair quality |
| [**Gathariki et al**](https://www.thieme-connect.de/products/ejournals/html/10.1055/s-0040-1715982) | 3 | 2 | 1 | 1 | 2 | 2 | 6 | 5 | Good quality | Fair quality |
| [**Khatib et al**](https://journals.lww.com/annalsplasticsurgery/Fulltext/2015/08000/Plastic_Surgery_Undergraduate_Training__How_a.20.aspx) | 1 | 2 | 0 | 1 | 2 | 2 | 3 | 5 | Fair quality | Fair quality |
| [**Singh et al**](https://link.springer.com/content/pdf/10.1007/s00238-022-02000-w.pdf?pdf=button) | 1 | 1 | 0 | 0 | 2 | 2 | 3 | 3 | Fair quality | Fair quality |

*A commentary on the quality of the studies was rated using the following scoring algorithm: ≥6 points were considered “good quality”, 2-5 points were considered “fair quality” and ≤1 point was considered as “poor quality”.*

Appendix 3

| **Influences subcategories** | |
| --- | --- |
| **Media** | Television, shows, social media, web research, internet (5) |
| **Teaching/university** | Clinical rotation, lectures/ teaching, societies (3) |
| **Personal exposure** | Previous exposure, personal experience, gap year, elective course (4) |
| **Independent mentorship** | Independent mentorship/shadowing (1) |
| **Interaction with others** | Doctors/professors, friends (2) |
| **Total** | 15 unique types of influences |
